# Food Gels Based on Polysaccharide and Protein: Preparation, Formation Mechanisms, and Delivery of Bioactive Substances

**DOI:** 10.3390/gels10110735

**Published:** 2024-11-13

**Authors:** Yong Guo, Chao Ma, Yan Xu, Lianxin Du, Xin Yang

**Affiliations:** 1 College of Sports and Human Sciences, Harbin Sport University, Harbin 150008, China; xuyan@hrbipe.edu.cn; 2 School of Medicine and Health, Harbin Institute of Technology, Harbin 150001, China; mmmmachao1996@163.com; 3 School of Chemistry and Chemical Engineering, Harbin Institute of Technology, Harbin 150001, China; 4 Graduate School, Harbin Sport University, Harbin 150008, China; dulianxin@hrbipe.edu.cn; 5 Shandong Benefit Mankind Glycobiology Co., Ltd., Weihai 264200, China

**Keywords:** food gels, protein, polysaccharides, bioactive substances, delivery systems

## Abstract

Hydrogels have a unique three-dimensional network that can create a good environment for the loading of functional compounds; hence, they have considerable potential in the delivery of bioactive substances. Natural macromolecular substances (proteins, polysaccharides) have the features of low toxicity, degradability, and biosafety; thus, they can be employed in the manufacture of hydrogels in the food sector. With its customizable viscoelastic and porous structure, hydrogels are believed to be good bioactive material delivery vehicles, which can effectively load polyphenols, vitamins, probiotics, and other active substances to prevent their influence from the external environment, thereby improving its stability. In this research, the common raw materials, preparation methods, and applications in the delivery of bioactive elements of food gels were examined; this study aimed at presenting new ideas for the development and utilization of protein-based food gels.

## 1. Introduction

A hydrogel is a hydrophilic substance consisting of a three-dimensional mesh of monomer or polymer chains that can absorb substantial quantities of water without disintegrating. Hydrogels can be generated through several techniques, usually via covalent or non-covalent cross-linking. Hydrogels can interact with water or biological fluids due to the equilibrium of osmotic and water-sum forces, resulting in the growth of network chain segments. This trait imparts hydrogels with special characteristics, including specific mechanical properties, solubility, and internal transport capacities. Consequently, hydrogels can be utilized across various sectors, including food packaging, pharmaceuticals, wound care, and tissue engineering.

With both liquid and solid characteristics, food gels are significant in the food industry. They are popular in the market because of their high water content, low calories, and great satiety [1,2,3]. Natural biopolymers are frequently employed as raw materials in the production of food gels to ensure their biosafety. Polysaccharides and proteins are naturally occurring macromolecules ubiquitous in food, serving as the fundamental structural and functional components. Polysaccharides possess significant thickening, gelling, emulsifying, and dispersion properties and are extensively utilized as texture modifiers in the food industry [4,5]. Polysaccharides commonly employed in food gel formation include chitosan, starch, and alginate. Compared to polysaccharides, proteins demonstrate a relative inadequacy in thickening and gelling properties, requiring higher mass fractions for gelation [6]. Proteins exhibit significant interfacial activity and can be utilized as emulsifiers and foam stabilizers in food processing, imparting distinctive textural qualities to food systems. Proteins include a higher quantity of functional groups for modification (e.g., amino, sulfhydryl, hydroxyl, and carboxyl groups) and demonstrate increased sensitivity to external environmental influences (e.g., pH, temperature). The functional groups of the protein’s own structure, including carboxyl, amino, hydroxyl, and sulfhydryl groups, give several possible applications for hydrogels in the food sector. And they can function as effective chemical reaction sites, provide target-specific delivery of bioactives, increase absorption and digestion of nutrients, are compatible with other species, and have good degradability. Common protein components include soy protein, pea protein, casein, whey protein, and gelatin. Numerous polysaccharides and proteins exhibit various architectures and physicochemical properties. Nevertheless, the rheological properties of a singular system are quite straightforward, complicating the attainment of accurate control over the gel food structure in practical applications. In recent years, the amalgamation of two or more polysaccharides or proteins has emerged as a prominent focus in the development of food gel structures. Unlike single gels, protein composite gels may effectively adjust gel texture and increase the diversity of gel designs [7,8]. Among them, biphasic hybrid polymer systems are the most basic and simple hybrid polymer systems. Biphasic hybrid systems encompass three categories: polysaccharide hybrid systems, protein hybrid systems, and polysaccharide–protein hybrid systems. Polysaccharide and protein hybrid systems demonstrate superior potential for the advancement of food gel structures compared to the first two hybrid systems. For example, by introducing hybrid systems of proteins and polysaccharides, multi-scale and multi-gradient composite gel structures can be produced [9,10], which depend on the interaction between the two polymers, the gelation mechanism, and the formation conditions [11]. In addition, as proteins are essentially natural amphiphilic macromolecules, the inclusion of proteins can further construct food gel forms comprising lipophilic components. The stable network structure of composite gels can effectively encapsulate unstable and decomposable bioactive compounds such as polyphenols and vitamins [12,13,14] to enhance the physicochemical stability and bioavailability of these functional chemicals [3,15]. This study provides a comprehensive analysis of the formation mechanisms and manufacturing methods of common composite hydrogels and outlines recent breakthroughs in the application of composite gels for the delivery of bioactive compounds.

## 2. Composite Gels with Polysaccharides and Proteins as Carriers

### 2.1. Polysaccharides Commonly Used in Complex Systems

Polysaccharides come from a wide range of sources, such as plant cell walls (e.g., cellulose, pectin, and peptidoglycan), animals (e.g., chitin, hyaluronic acid, and chondroitin), and microorganisms (e.g., xanthan gum and dextran). They are widely used in biomedicine because of their good water absorption, biocompatibility, and degradability [16]. The primary chains of polysaccharides contain numerous carboxyl, hydroxyl, amino, and aldehyde groups, capable of forming gels via physical interaction (such as hydrogen bonding, electrostatic interactions, and hydrophobic interactions) or through chemical cross-linking [17,18]. Different formation pathways confer distinct functional features to polysaccharide hydrogels, including self-healing, temperature sensitivity, and adhesion [19]. Polysaccharides may impact many physiological systems in the body and possess therapeutic capabilities, including immunomodulation, anti-infection, anti-tumor, and anti-hypoglycemic actions.

#### 2.1.1. Chitosan

Chitosan is a prevalent component utilized in the formulation of natural hydrogels. Chitosan is a cationic linear polysaccharide composed of N-acetyl-D-glucosamine and D-glucosamine units, synthesized through the deacetylation of chitin [20]. Chitosan is hydrophilic and can be biodegraded by lysozyme, acid, and colon bacteria, making it biocompatible and biodegradable. It can be used as a raw material for biomedical materials [21]. The formation of physically crosslinked chitosan hydrogels is based on secondary forces through non-covalent bonds such as hydrophobic interactions, hydrogen bonding, ionic/electrostatic interactions, crystallization, polyelectrolytes, and steric complexation, which were developed via a method of freezing–thawing accompanied by the addition of different concentrations of honey to the hydrogels in the study of Lavanya. The hydrogels consisted of chitosan, polyvinyl alcohol (PVA), and gelatin in a 2:1:1 (*v*/*v*) ratio, demonstrating a highly porous and spongy structure similar to the extracellular matrix of skin. The integration of honey into polyvinyl alcohol (PVA)/chitosan/gelatin hydrogels demonstrated no signs of toxicity. The hydrogels infused with honey demonstrated increased cell growth rates in MTT assays. The H-10 group demonstrated the greatest biocompatibility. The addition of honey led to decreased mechanical qualities; nonetheless, the samples demonstrated the essential tensile strength and viscoelasticity for skin tissue and degraded adequately to enable wound healing [22]. Chitosan hydrogels were manufactured employing chemical cross-linking in the presence of cross-linking agents for enhanced chemical stability. The stability of chitosan hydrogels was increased. Various chemical reactions are employed to establish covalent bonds within the hydrogel network, including free-radical polymerization, addition and condensation polymerization, Diels–Alder click chemistry, Schiff base reaction, oxime synthesis, enzyme-induced cross-linking, and photo-cross-linking [23].

#### 2.1.2. Starch

Starch is among the most ubiquitous natural biopolymers in existence. It is characterized by its renewability, biodegradability, and biocompatibility. Starch granules are particles characterized by crystalline and amorphous regions, primarily composed of linear and branched chain starch [24]. Straight-chain starch contains a linear structure and comprises glucopyranose units united by α-D-(1-4) glycosidic connections. Branched-chain starches have a branched structure in which the linear branched chains are related through α-D-(1-4) glycosidic bonds and periodically through α-D-(1-6) glycosidic linkages [25]. When starch granules are cooked in water, a thick starch paste is created through a dextrination process. Upon cooling, straight and branched chain starches re-establish an orderly structure known as retrogradation [26]. During retrogrowth, high-concentration starch pastes typically yield starch hydrogels, whereas low-concentration starch pastes result in precipitation. Common methods for the fabrication of starch-based hydrogels by physical processes include starch regrowth and extrusion [27]. Zeng et al. investigated the printability of different ratios of maize starch and rice starch at various temperatures. A suspension of maize starch and rice starch (3% *w*/*w*, dry basis) was initially cooked in a 65 °C water bath for 20 min. After cooling to 25 °C, starch was added to obtain 10%, 15%, 20%, 25%, and 30% (*w*/*w*, dry basis) corn starch and rice starch suspensions. The starch suspensions were subsequently utilized to formulate starch hydrogels at varying temperatures (65, 70, 75, 80, and 85 °C). The findings indicated that the corn starch hydrogel concentration was 20% at a printing temperature of 70–75 °C, while the rice starch hydrogel concentration ranged from 15% to 20% at a printing temperature of 75–80 °C and exhibited commendable mechanical strength and extrusion characteristics, suggesting their suitability for 3D printing via thermal extrusion [28]. Chemical cross-linking involves the employment of cross-linking chemicals to unite two or more starch chains or graft monomers onto the starch backbone. Citric-acid-crosslinked-starch-based hydrogels and polymer-grafted-starch-based hydrogels are two extensively researched chemically modified hydrogels. Citric acid is frequently utilized as a cross-linking agent in the production of starch hydrogels. Yusof et al. created crosslinked carboxymethyl sago starch-based hydrogels by employing esterification of the carboxyl group of citric acid with the hydroxyl group of carboxymethyl sago starch. The chemically crosslinked starch hydrogels displayed better stability than carboxymethyl sago starch. The thermogravimetric analysis revealed that the hydrogel’s peak decomposition temperature was 301.15 °C, whereas carboxymethyl sago starch had a maximum decomposition temperature of 284.7 °C [29].

#### 2.1.3. Pectin

Pectin is a naturally occurring polysaccharide found in plant cell walls, mainly consisting of repeating units of *α*-(1-4)-linked D-galacturonic acid. The pectin structure may consist of high galacturonan (HG), rhamnogalacturonan I (RG-I), and rhamnogalacturonan II (RG-II) structural domains, depending on the plant source [30]. This compositional heterogeneity allows pectins to exhibit unique properties, such as their gelation, solubility, and rheological behavior. Pectins may form three-dimensional networks of hydrophilic polymer chains, making them appropriate for hydrogel formation. Highly methylated high-fat pectins primarily gel through hydrophobic cohesive forces and the formation of hydrogen bonds under particular environmental conditions. Low-methylated (DM) low-ester pectins possess a higher number of free carboxylic acid groups, allowing for the creation of a continuous gel network through interactions with Ca^2+^ in an “egg carton” arrangement [31]. Popov et al. developed hydrogels utilizing ionic gelation using low-methylated apple and pigweed pectin samples, containing calcium gluconate. The procedure for hydrogel preparation is as follows: Ionic hydrogels are formed when polymers gel in the presence of metal cations. Pectin and its carboxylic acid groups readily form gels in the presence of metal cations, such as calcium ions (Ca^2+^). Calcium gluconate is a divalent metal cation that interacts with pectin, enhancing its gelling properties. Calcium gluconate is favored over calcium chloride due to its more appealing flavor, boosting market penetration. Furthermore, sucrose creates hydrogen bonds with water molecules, resulting in the immobilization of free water and boosting the concentration of the polymer environment, therefore stimulating gelation. This work indicated that a combination of apple and pigweed pectin exhibited a synergistic impact that boosted the gel strength of hydrogels generated by ionic gels [32].

### 2.2. Natural Proteins in Complex Systems

Natural food proteins, including both plant and animal sources, have excellent gel properties and can be exploited to generate food gels [33], for instance, the bulk of plant proteins, whey proteins, and comparable compounds. These proteins can undergo gelation when treated to extreme temperatures. At sufficiently high concentrations, heat causes the protein chains to unroll, twist, and assemble, creating thermally irreversible gels [34,35]. In rare cases, elevated pH and ionic strength can denature proteins, leading to gel formation. The distinctive amino and carboxyl groups in the protein structure can also facilitate the creation of gels by physical/chemical cross-linking [35]. The amino acid composition, sulfur-containing amino acid content, molecular weight, protein subunits, and surface hydrophobicity of proteins influence their gelling behavior and gel structure [36]. Protein gels are highly biocompatible, do not induce immune responses, are easy for cellular interactions as well as good degradability, and have a wide range of applications in biomedicine and food processing [37,38] (as shown in Table 1).

#### 2.2.1. Soy Protein Gel

**Figure 1:**
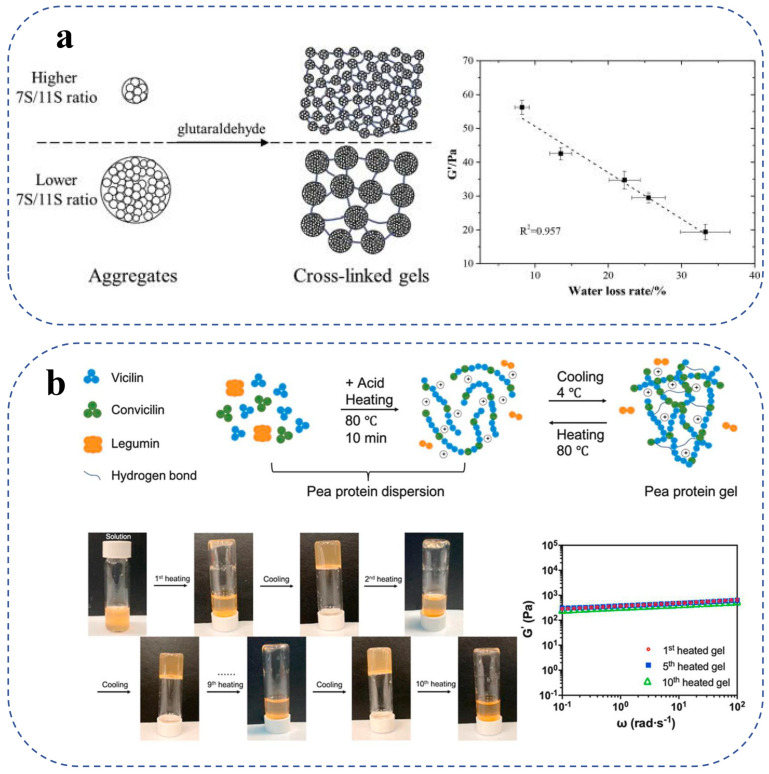
(**a**) Schematic diagram of the gel network formed by different-sized aggregates of glutaraldehyde-cross-linked soy protein, forming a transparent gel of pea protein isolate [39]. (**b**) Schematic diagram of the preparation of pea protein isolate by thermos-reversible gel formation process [42].

Soybean isolation protein (SPI) implies a highly purified and concentrated version of soy protein obtained from soybeans. Soy protein isolate (SPI) is often produced by crushing soybeans into fine flakes and removing fat using hexane. The proteins in SPI are classed into 2S, 7S, 11S, and 15S proteins based on their sedimentation coefficients following high-speed centrifugation [52]. 7S (*β*-conglycinin) and 11S (glycine) are the major components, collectively totaling around 70% (*w*/*w*) [53]. 7S is a trimeric glycoprotein consisting of three subunits, *α*, *α*′, and *β*, whereas 11S is a hexameric glycoprotein, with each of the 11S subunits consisting of an acidic (A) polypeptide and specialized basic (B) polypeptides joined by disulfide bonds. Soy proteins can be transformed into a gel through the application of acids (e.g., acidifiers and lactic acid bacteria), heat, enzymes, and salts [54,55,56]. Wang et al. showed that heating soy proteins to 100 °C for 30 min at protein concentrations of up to 6% (*w*/*v*) can efficiently produce soy proteins with enhanced gel characteristics [57]. In contrast, the gelling properties of soy protein gels decrease as the protein’s particle size decreases and its mobility rises at lower protein concentrations. Wu et al. demonstrated that, following the same preheating process, protein compositions with different 7S/11S ratios resulted in the formation of aggregates with varying particle sizes, as shown in Figure 1a. Because of their larger pores and rougher gel networks, larger particles generated with lower 7S/11S ratios showed higher gel dehydration rates [39]. Chemical modifications, such as glycosylation, phosphorylation, and acylation, can also occur in soy proteins. Gu et al., for instance, added reducing sugar to the tofu-processing process and heated SPI over its denaturation temperature at neutral pH. They then used glucono-δ-lactone GDL to induce gelation at isoelectric pH and heat-treated the acidic gel once again. While SPI heat treated with sucrose was not glycosylated, SPI heat treated with glucose at neutral pH had a higher degree of glycosylation than lactose. At neutral pH, glucose and lactose prevent proteins from denaturing, resulting in GDL-induced gels that have a higher water-holding capacity and a lower gel hardness [40]. He et al. reported a decrease in Gʹ of SPI gels made by succinic anhydride acylation, which may be related to the soy protein’s decreased hydrophobicity after acylation [41]. Guo et al. used hexametaphosphate or sodium tripolyphosphate to modify SPIs by industrial phosphorylation to modify SPI. Rheological results showed that the G′ and G″ of emulsion gels increased and exhibited significant elastic behavior after SPI phosphorylation [58].

#### 2.2.2. Pea Protein Gel

In addition to soybeans, peas are a popular legume protein used in the food business that has attracted a lot of attention [59]. Depending on the type and environment, peas have a high protein content, ranging from 20% to 30% [60], which makes peas one of the protein-enriched sources that can provide better sustainability and proper nutritional value and functionality [61]. The significant nutritional value, hypoallergenic qualities, and non-GMO designation of pea proteins make them notable [59]. The two main proteomes found in pea proteins are albumin and globulins, which make up 10–20% and 70–80% of the total, respectively. The three primary proteins of globulins are legumin (11S), vicilin (7S), and convicilin. Legumin is a hexameric protein (approximately 360 kDa) with six subunits connected by disulfide bonds [62], while vicilin and convicilin are trimeric proteins with respective molecular weights of approximately 150 kDa and 210 kDa that do not contain disulfide bonds [63]. Chen et al. described a technique for creating thermos-reversible protein gels from pea isolates that were recovered via ammonium sulfate precipitation, as shown in Figure 1b. With a pH range of 2.4 to 4.2 and a protein concentration of 10 to 15%, thermos-reversible gels were created. They showed good mechanical properties at a pressure of 6.32 ± 1.40 kPa. The thermos-reversibility of the gels was demonstrated to be stable after repeated heating and cooling from 80 °C to 4 °C by dynamic rheological studies. According to scanning electron microscopy (SEM), clear gels with a fine-chain network formed more readily at lower pH values. As the pH rose to 4.2, the gels’ transparency dropped, but their thermal reversibility stayed constant even as the gel network broke up into particle aggregates. According to the findings, disulfide bonds and hydrophobic contacts have a minor role in the creation of the gel network structure during cooling, but hydrogen bonding predominates. Pea protein gels are transparent, thermally reversible, and have good mechanical qualities, making them a viable alternative to gelatin in food applications such as fermented foods, fruits, and drinks with slightly acidic pH levels [42]. Chen et al. used protein conformation studies, gel rheology, and solubility tests to thoroughly investigate the effects of high pressure processing (HPP), treatment duration, protein concentration, and sample pH on the microstructure and strength of pea protein gels. At room temperature, pea protein had a minimum gel-forming concentration of 10–15% (*w*/*v*) at 600 MPa and 300 MPa of pressure. The microstructure and properties of HPP-treated gels were strongly impacted by the pH. Hydrophobic interactions were the main stabilizing factor for particle gels with a dense structure and a mechanical strength of up to 130 kPa at pH 5. On the other hand, the fine-chain gels that were created at pH 7 showed remarkable flexibility and a significant capacity to retain water. These gels were primarily maintained by hydrogen-bonding interactions. To produce a range of pea protein gels that mimic the textures of tofu, pudding, and jelly goods, the pH, HPP pressure level, and duration were changed. Because of HPP’s adaptability, pea protein can be used as a gelling agent in a range of culinary applications in place of soy or animal proteins. Furthermore, gels treated with HPP after pH conversion showed excellent freeze–thaw durability with a minimal dehydration shrinkage level of 15% after two freeze–thaw cycles, indicating their potential for use in frozen meals [43].

#### 2.2.3. Corn Alcohol Protein Gel

With an average molecular weight of 25–45 kDa, zearalbumin is a naturally occurring, macromolecular, biodegradable, non-allergic, and “generally recognized as safe” amphiphilic alcohol soluble protein. Its four constituents—*α*, *β*, *γ*, and *δ*—have varying molecular sizes, solubilities, and peptide chains [64]. Among these components, *α* protein accounts for 70–85% of the total composition of zeinolysin [65]. Although zeinolysin is insoluble in water, it is soluble in 40–90% ethanol [66]. Additionally, it dissolves in organic solvents like acetic acid, propylene glycol, sodium dodecyl sulfate (SDS), strong bases, and high urea concentrations. Under some conditions, corn alkyd soluble proteins can form gels on their own. It was demonstrated that purified *α*-zearalolysin could gel by close packing at high concentrations and partially dissolve in aqueous ethanol solution; zearalolysin could also self-assemble at greater aqueous acetic acid levels. When kept at room temperature, the 50% (*w*/*v*) zeinolysin is evenly distributed and forms gels that sustain themselves and have solid-like qualities. To increase the gel stability and water retention of their gels, corn alcohol soluble proteins can also be altered or covalently crosslinked. Carboxyl groups with adjustable hydrophilic/hydrophobic characteristics can be added to amide alcohol-soluble proteins by using citric acid or acetic anhydride [44]. Hydrogels with crosslinked networks that dissolve in phosphate buffer solutions and pure water are produced when sodium hexametaphosphate phosphorylates unreacted hydroxyl groups or N-terminal amino groups in zeinolysin. Additionally, SDS can change the structure of zeinolysin, add polar groups and negative charges, and use chelation to generate a strong and stable adhesive with Fe^3+^ [45]. By introducing glutaraldehyde cross-linking via covalent bonds, the Schiff reaction also transforms zeinolysin into anionic protein nanoparticles, which are subsequently fixed in the porosity network of a chitosan-poly(vinyl alcohol) gel. Furthermore, the gel network containing the nanoparticles forms a stronger and more compact structure as a result of the free -OH and -NH_2_ imine bonding [67].

#### 2.2.4. Whey Protein Isolate Gel

Whey protein isolate (WPI) is a by-product of cheese production. It is composed of many globular proteins, such as beta-lactoglobulin (beta-LG), alpha-lactalbumin (alpha-LA), bovine serum albumin (BSA), and lactoferrin (LF), and it usually has a protein content of over 90%. WPI’s high nutritional content and favorable physical and chemical characteristics, such as its capacity for foaming, emulsifying, film-forming, and gelling, make it a popular ingredient in food preparation [47]. Bovine serum albumin (BSA) and *β*-LG are the gelatinous proteins found in whey protein isolate. The most popular technique for gelatinization is heat-induced gelatinization, which creates heat-induced gel by heating denaturation aggregates and then cooling them with an ice water bath. Whey protein isolates undergo heat denaturation, which opens their internal structure. Intermolecular condensation then takes place to create a gel with a three-dimensional network structure [68]. Strong water retention is achieved via the gel network’s pore structure, which can trap in water and other substances. Protein concentration, pH level, ionic strength, polysaccharide type and concentration, and other factors were used to evaluate the gelling temperature and gelling characteristics of whey protein isolate gels [69]. Whey protein isolate thermal gel has a comparatively modest temperature, typically above 65 °C. When whey protein isolate molecules are heated, they denaturize, their internal hydrophobic structure expands, their hydrophobic groups become visible, and a thermally irreversible gel is created through cross-linking brought on by the interchange of sulfhydryl and disulfide bonds. Whey protein isolates have an isoelectric point between 4.5 and 5.5. Irreversible gels are also produced when the system’s pH is significantly higher than the whey protein isolate’s isoelectric point. A reversible gel is created when the pH is below the isoelectric point of the whey protein isolate because the electrostatic force between protein molecules is stronger, and the disulfide bond interaction is weaker. Mehdi Mohammadian et al. heated a whey protein isolate solution with a pH of 5.9 to create curcumin-loaded whey protein isolate microgels. Curcumin was injected into the whey protein microgel in an amorphous form, according to XRD measurements. Curcumin and whey protein microgels simultaneously exhibited hydrophobic interactions and hydrogen bonding, according to the results of FT-IR and fluorescence spectra. When curcumin was included into whey protein microgel, its antioxidant activity and sedimentation were greatly enhanced. According to the study, whey protein microgels are a viable and efficient way to add curcumin to functional foods, which have improved health-promoting qualities [70].

### 2.3. Composite Gel

Single hydrogels exhibit drawbacks like inadequate gel strength, restricted water retention capacity, and susceptibility to photo-thermal instability [71]. Multicomponent composites are commonly used to develop customizable gel structures in order to overcome the current limitations of single gels. Several studies have shown that protein–protein and polysaccharide–polysaccharide composite gels successfully improve the properties of single-component gels. Researchers are becoming more interested in the polysaccharide–protein combination that creates gels with distinct functional differences and different chemical structures.

#### 2.3.1. Protein–Protein Composite Gel

**Figure 2:**
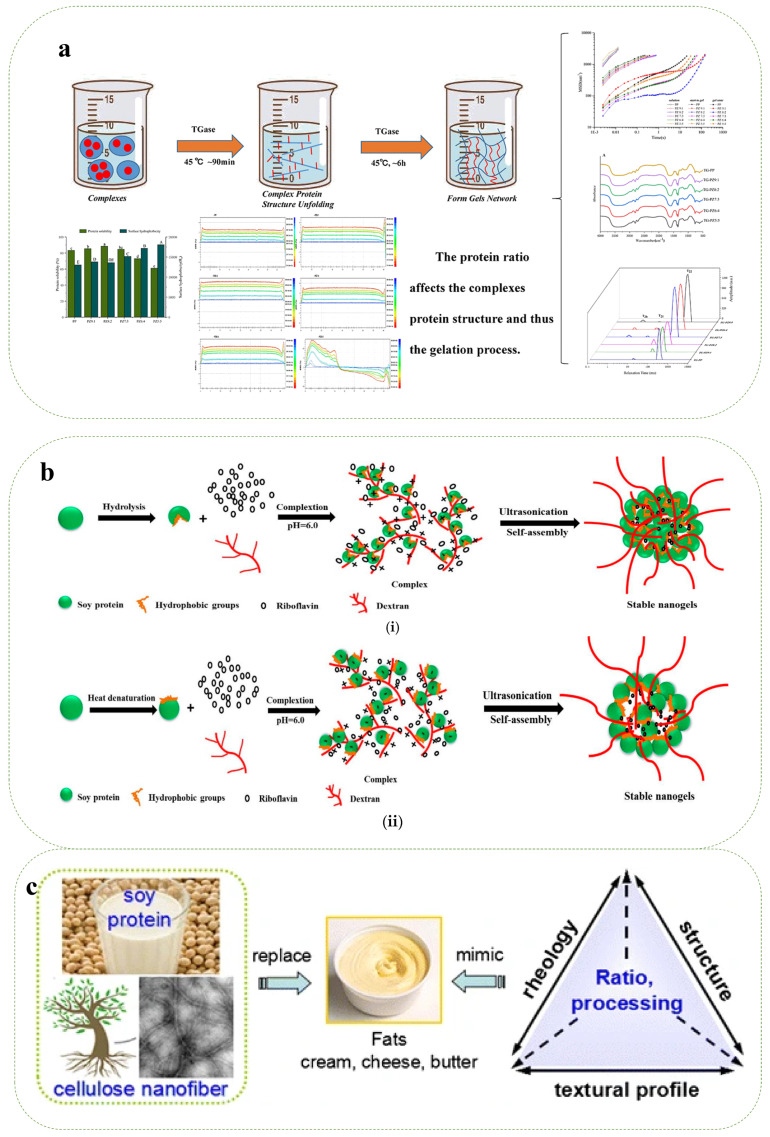
(**a**) Schematic representation of TGase-induced gel characterization of pea-protein/zeatin complexes [48]. (**b**) Schematic representation of ultrasound-induced self-assembled modified soy protein/dextran nanogel as a delivery vehicle for riboflavin [50]. (**c**) Schematic representation of soy isolate protein/cellulose nanofibrous composite gels prepared as a fat substitute [53].

Binary protein composite gels are a relatively new field of study, but interactions between single-component proteins have been extensively studied [72]. Since individual proteins, especially hydrophobic proteins, have poor dispersion and are more prone to aggregation, hydrophobic forces are usually the main driving mechanism when many proteins interact [73]. Liu et al. used a thermal enzyme-induced approach to manufacture WPI–gelatin composite hydrogels using gelatin and whey proteins as basic ingredients. The WPI–gelatin mixture was heated above the whey protein’s thermal denaturation temperature for thermally induced gelation, which caused the protein chains to unfold for cross-linking. By cross-linking gelatin and heat-denatured whey protein in the WPI–gelatin combination, glutamine transferase was added to aid in enzyme-induced gelation. By changing the electrostatic interactions between the proteins, the initial pH of the WPI solution influences the thermal aggregation of proteins, which in turn influences the macroscopic properties of the hydrogels that are created. On the other hand, compared to the thermally produced hydrogels, the enzyme-induced hydrogels solidified more quickly and had much greater elastic moduli at pH 7 (2566 Pa) and pH 8 (1566 Pa). While the heat-induced hydrogels showed better freeze–thaw resilience, the enzyme-induced hydrogels also showed greater stiffness and swelling. By altering the pH and manufacturing procedure, hydrogels’ structure and texture can be changed, expanding their uses and aiding in the creation of novel food products [47]. As shown in Figure 2a, Liu et al. used a pH-based method to combine different mass ratios of pea proteins and maize alkyd proteins (10:0 to 5:5) into nanoparticles. They next used TGase to help the composite protein particles gel. By using the Maillard process, the study assessed how varied protein mass ratios affected the creation of composite gels and their properties. At a 20% concentration of maize alkyd soluble protein, the composite gel had the highest gel strength (145.39 ± 1.34 g), water retention capacity (93.21 ± 1.59%), and slow-release properties. The quantity of disulfide bonds in the system is altered by the varied protein mass ratio, which can effectively control the gel’s strength. The structure of complex proteins and their functional characteristics can be modified by adjusting the protein mass ratio, providing a fresh approach to enhance gel properties [48].

#### 2.3.2. Protein–Polysaccharide Composite Gels

Various types of polysaccharide gum and protein have various formation mechanisms, which leads to diverse gel structures and, eventually, different gel characteristics. The thermal denaturation and stretching of proteins, their aggregation, and the cross-linking of aggregates to produce gel are generally accepted as the colloidal mechanism of proteins and polysaccharides. Protein–polysaccharide interactions, including as hydrophobic, disulfide, hydrogen, and electrostatic interactions, are the primary cause of the aggregation of proteins and polysaccharides to produce gel. For instance, electrostatic interaction can generate gels when proteins and polysaccharides have opposite charges. The electrostatic interaction between polysaccharide and protein is impacted by varying ionic strength, which in turn impacts gel formation. Protein and polysaccharide aggregation, decreased electrostatic repulsion, enhanced crosslinking strength, and partial charge shielding can all result from high salt ion strength [8]. Yu et al. ultrasonically dissolved cellulose nano-fibrillated CNF from wood flour and used it in conjunction with soy protein SPI as a rheology modifier and structural stabilizer, as shown in Figure 2c. The resulting SPI–CNF mixtures had a higher modulus and more viscosity. At a 7:1 SPI/CNF ratio, the mixture’s TPA properties were quite similar to those of cream. By substituting 10% of the cream with this mixture, the desired effects of lower fat, low calories, melt resistance, and a similar texture and flavor were achieved [49]. Modified soy protein/dextran nanogels (NGs) were created by Qiu et al., using a straightforward self-assembly technique triggered by ultrasound, as seen in Figure 2b. Utilizing FTIR spectroscopy and XPS studies, the establishment of NG was confirmed. The modified soy protein/dextran nanogel exhibited a nearly spherical core–shell form in contrast to the hydrodynamic soy protein–dextran nanogel (NG1), which had a diameter of roughly 138 nm. The nanogels held up well in a variety of environmental circumstances. The addition of riboflavin to the nanogels caused very little change in their particle size. At a riboflavin concentration of 250 μg/mL, the nanogel’s encapsulation efficacy reached 65.9%. In simulated intestinal fluid (SIF) as opposed to simulated gastric fluid (SGF), the nanogels showed a faster release rate. According to the findings, modified soy protein–dextran nanogels show promise as a delivery system for medications and bioactive compounds [50]. Yin et al. created curcumin (Cur@ZA)-loaded zeinolysin–alginate “core–shell” structures using an anti-solvent precipitation approach. Zeinolysin served as the “core”, while alginate gels were formed as “shells” using calcium-induced gelation. Through hydrogen bonding, hydrophobic interactions, and electrostatic adsorption, Cur@ZA’s zeolysin “core” and alginate gel “shell” are firmly joined. Curcumin outperformed 92% in terms of encapsulation efficiency in the Cur@ZA nanogel. During the simulated digestion process, the addition of calcium ions facilitated the slow and steady release of curcumin and improved its photostability and thermal stability. Therefore, this novel nanogel delivery technique has the potential to be used in the food business and has favorable physicochemical characteristics, stability, and controlled-release capabilities [51].

## 3. Preparation Methods and Interaction Mechanisms of Composite Gels

The amalgamation of polymer dispersion or dispersive solution, which is influenced by external environmental factors like temperature and pressure, results in gel formation. Based on the different contact forces, it can be divided into chemical and physical gels. On the other hand, non-covalent interactions support the three-dimensional network architecture of physical gels. Gels are created by hydrophobic, hydrogen-bonding, or electrostatic interactions, which generate a certain structure with internal spaces. Water may be divided into bound and free water within the polymer network as a result of possible interactions with the gel structure. Gels are being used as delivery systems for bioactive substances, biomacromolecules, and encapsulated nanometric molecules. Depending on the characteristics of the gel, bioactives can be adsorbed onto the surface, covalently bound to the backbone, encapsulated internally, or dissolved inside the matrix [74]. As a result, food gels may have a number of uses in the delivery of bioactive compounds.

### 3.1. Interaction Mechanisms of Gels

The two biomacromolecules’ various chain segments and side chains interact with one another by a variety of molecular interactions, including electrostatic interactions, solvent repulsion, valence bonds, hydrogen bonds, ionic bonds, hydrophobic interactions, and van der Waals forces [75]. Different types of intermolecular contacts constitute powerful forces that propel self-assembly and create stable network architectures, despite the weakness of single-molecule connections. In three phases, polysaccharide–protein complexes are formed when proteins and polysaccharides mix in solution. Electrostatic interactions are the main driving factor in the first step, which is a rapid and erratic primary complexation process [76]. A Coulombic force, or simply a strong interaction, is what the electrostatic interaction is. Electrostatic forces are the main factor influencing polysaccharide–protein complexes [77]. The methodical creation of complex systems is the focus of the following stage. The formation of chemical bonds reorganizes the internal structure into a specific shape. The aggregation phase of polysaccharide–protein complexes is the third stage. Hydrophobic interactions are the primary cause of rearrangement in complex aggregation [78].

Proteins and polysaccharides are usually found in watery settings as charged substances. Therefore, depending on the macromolecular charge and the surrounding environment, polysaccharides and proteins form complexes through two different types of interactions: thermodynamically compatible and thermodynamically incompatible [79]. The two most significant ways that polysaccharides and proteins interact through are mutual attraction and repulsion. In various phases, the complex system has varying effects on the functional characteristics of protein and polysaccharide complexes. When building polysaccharide–protein nanocomposites, the two main interactions that need to be considered are thermodynamic compatibility and incompatibility. Proteins and polysaccharides’ thermodynamic compatibility is mostly determined by their charge properties. Physical and chemical characteristics, such as molecular weight, molecular structure, charge distribution, charge capacity, and molecular chain flexibility, all affect thermodynamic compatibility. Depending on the surrounding conditions (such as pH, temperature, ionic species, ionic strength, and other elements), polysaccharides, proteins, and polyelectrolytes can display a range of physical and chemical characteristics. For improved loading and transport of different elements, gels must be made using the properties of proteins and polysaccharides.

### 3.2. Preparation Method of Gel

#### 3.2.1. Physical Methods

##### pH-Induced Gelation

The pH of the solution can modify the solubility, molecular structure, and charge density of polysaccharides and proteins, etc. Consequently, the inter- and intramolecular interactions of polysaccharides or proteins are frequently modified by varying the pH during the formation of composite gels [80,81]. When pH > pI, proteins acquire a negative charge, resulting in the phase separation of neutral or anionic polysaccharides, preventing gel formation; conversely, when the pH is below pI, proteins become positively charged, enhancing electrostatic attraction, thereby facilitating gel formation between proteins and polysaccharides [82]. pH-induced methods do not require the inclusion of organic solvents and chemical treatments, and they are a simple, safe, and economical way for the manufacture of gels [83]. For example, at pH 7, the gelation temperature (T gel) of coupled proteins, such as serum albumin and oppositely charged lysozyme, is lower than the melting temperature (Tm) of the individual proteins. Since these proteins are mostly *α*-helical, it is likely that electrostatic forces caused the observed gels. All proteins acquire a homogenous charge and show Tgel < Tm when the pH of the solution changes to an acidic or basic level, which is attributed to pH-induced denaturation [84]. pH induction is a cost-effective, simple, and safe method.

##### Heat-Induced Gelation

Heat-induced gelation is a popular method for creating composite hydrogels since it does not require chemical initiators or crosslinking agents, making it a preparation process that is safe for the environment. Proteins denaturize, unfurl, and aggregate to create a gel when heated [85]. For example, a 1% (*w*/*w*) sodium dodecyl sulfate (SDS) solution, N-ethylmaleimide (NEM) solution, and whey protein powder dissolved in water can all be heated to 80 °C for 30 min or 85 °C for 60 min to start the gelation process. According to rheological and compressive studies, the degree of disulfide bonding in WPC gels increases their rubbery nature (rupture strain), while the degree of non-covalent bonding between denatured protein molecules increases the gel’s stiffness (modulus) [86]. The structure and properties of thermally produced hydrogels are significantly influenced by the heating temperature and time.

##### Cold-Induced Gelation

The conditions for cold-induced production of composite hydrogels are less harsh than those for heat-induced formation; however, the growth of composite gels requires the presence of other ingredients like cross-linking agents, salt ions, or acids [71]. When protein solutions are dissolved at neutral pH (above the isoelectric point), below the degree of protein gelation, and heated with low ionic strength, cold-induced protein gels can be produced. In these conditions, globular proteins partially unfold and become denaturated. To achieve the isoelectric pH of the proteins, salts or acids are added to the solution. This lowers inter-protein repulsion and makes it easier for the proteins to crosslink to form gels. At 4 °C, it was discovered that surimi lysate transformed into a semi-gel structure, showing reduced network density and aggregation in comparison to heat-induced gels. Protein cross-linking and storage conditions are the main factors that turn surimi lysate into a weak gel. The quality of chilled surimi lysate products is better preserved throughout production and storage thanks to this study [87]. By facilitating various interactions between proteins and bioactive compounds, cold-induced gels can create novel, targeted delivery systems.

##### Ion-Induced Gelation

It has been common practice to use salt ions like Na^+^, K^+^, Ca^2+^, Mg^2+^, Fe^2+^, and Fe^3+^ to promote the development of hydrogels. Composite gels are mostly formed in salt ion induction by electrostatic and hydrophobic interactions. By promoting droplet contacts and increasing protein aggregation, salt ions can produce a denser gel structure with superior water-retaining properties [88]. The nature and concentration of salt ions influence the production of composite gels. For example, divalent or high-valent ions are more effective than monovalent cations in limiting electrostatic attraction [89,90,91], and a low concentration of solvent is useful in reversing the inhibition of composite gel formation produced by the addition of salt ions [92]. The composite gels’ ability to retain water, gel hardness, crystallinity, and thermal stability are all improved by the addition of salt ions [93,94]. When monovalent and divalent ions are added during the yolk gel formation process, the secondary structure of the yolk proteins continuously shifts from the *α*-helix and *β*-shift to the *β*-folding, encourages hydrophobic aggregation between protein molecules, and strengthens the hydrophobic interactions between yolk proteins. These conformational changes encouraged the production of a dense regular mesh structure with smaller pores, which significantly increased the gel’s hardness, WHC, and viscoelasticity without significantly changing its color [95].

##### Enzyme-Induced Gelation

Hydrogels can be produced biologically by enzyme-induced gel formation. Enzymes can stimulate the formation of stable covalent bonds in segments of polymer chains under moderate conditions. As an example of enzyme-induced gel formation, transglutaminase promotes intramolecular and intermolecular cross-linking of peptide chains, which makes protein gelation easier. While the egg protein gelatinized and spread throughout the SPI gel network, the addition of TG crosslinked the SPI inside the egg–SPI composite solution, significantly improving the gelling properties of the egg–SPI composite gel. According to this work, TG is a useful method for increasing the hardness of egg–SPI composite gels without compromising their flexibility or ability to retain water [96].

#### 3.2.2. Chemical Methods

Chemical crosslinking methods to produce protein composite gels largely include metal coordination processes, certain chemical molecules, and biological crosslinking [97].

##### Metal-Ion-Induced Gelation

The formation of metal ion-induced gels fundamentally depends on the establishment of a network structure through electrostatic or coordination interactions between metal ions and polysaccharide molecules [98]. This structure then forms a network and creates an interpenetrating structure by interacting chemically or physically with other linear or polymer molecules. The plant polysaccharide ASKP can be crosslinked with Fe^3+^ to create a hydrogel. When combined at a 9:1 ratio at pH 4.0 with 60 mM Fe^3+^, the addition of branching starch or gelatin can significantly increase the composite hydrogel’s gel strength. ASKP complexes with gelatin or branched starch show excellent compatibility; through electrostatic interactions and hydrogen bonds, the ASKP chains interweave with the gelatin and branched starch. ASKP significantly increases gel strength when it interacts with branched starch or gelatin to generate a complex hydrogel with a thick, semi-interpenetrating network [99].

##### Organic-Compound-Induced Gelation

Unlike metal ions, chemically crosslinked gels are created when organic compounds interact with polymer chains. In order to create composite gels, the gels eventually interact physically with another polymer through entanglement and interpenetration. Glutaraldehyde and tri-functional polyurethane prepolymers are examples of common crosslinkers. Under normal pH conditions, gelatin solutions can form complexes with polysaccharides that have opposing charges. Commercial gelatin (Type B) has an isoelectric pH range of 4.8 to 5.0. The majority of the charges in the gelatin solution are negative when the pH is higher than the isoelectric point. As a result, charge-similar loquat gum CTG polysaccharides cannot form stable complex aggregates with gelatin solutions. When the crosslinker glutaraldehyde is added to a protein–polysaccharide mixture, imine connections are created, which results in the formation of a stable gel structure. The gelatin chains’ amino groups react with glutaraldehyde at pH 5.4 to form acetal connections, which causes chemical crosslinking and the formation of a network structure. The CTG–GA gel is produced when CTG interacts with gelatin chains via hydrogen bonding in the network structure [100].

##### Biological-Crosslinker-Induced Gelation

The mechanism of chemical molecules and biological crosslinkers work is similar. In both cases, the crosslinker reacts with proteins or polysaccharides to create chemically crosslinked gels. For instance, genipin is a naturally occurring biological crosslinker that is frequently used for biopolymers including chitosan, collagen, and gelatin. For instance, a novel injectable hydrogel was created using phycocyanin (PC), which is known to enhance diabetic wound healing and act as protein nanocarriers. It is composed of collagen, gelatin, chitosan, and genipin as a crosslinking agent. This technique uses genipin, which is naturally low-toxic, as a crosslinker. It is also highly biocompatible, 10,000 times less cytotoxic than glutaraldehyde, reduces host inflammation, and is more environmentally friendly. It is expected that adding chitosan and nanoparticles will improve the storage modulus, which will improve the hydrogel’s mechanical properties [101].

#### 3.2.3. Other Methods of Improvement

The gelling ability of proteins and polysaccharides can be changed by adjusting their concentration, temperature, and pH, which will change the molecular contact forces. This section provides a quick overview of a number of cutting-edge protein modification techniques meant to improve proteins’ gelling properties.

##### High Pressure Processing (HPP)

High pressure processing (HPP) is a developing food preservation method that involves the use of liquid as a medium in a sealed ultra-high pressure vessel to treat food products in the pressure range of 100–800 MPa [102]. Both covalent and non-covalent connections between protein molecules are disrupted by HPP therapy. During HPP, the number of disulfide bonds increased, while the number of free sulfhydryl groups in proteins decreased [103]. As the homogenization pressure increased, the gel network structure became more uniform. Additionally, HPP decreased protein solubility and increased surface hydrophobicity at high protein concentrations, which made it easier to form gel networks using WHC.

##### High-Intensity Ultrasound (HIU)

High-intensity ultrasound (HIU) alters the structure of materials by modifying their physical energy. Cavitation is created in a solution using ultrasonic pressure, which causes bubbles to expand and burst. Protein unfolding and reactive group exposure brought on by the released energy alters protein function. In order to create emulsified gels of inulin, carrageenan, and SPI, Paglariini et al. used a thermally induced method. Ultrasonography increased the hydrophobicity and solubility of the protein surfaces, which increased the gels’ hardness and water retention [104].

##### Microwaves

Microwaves, with frequencies ranging from 300 MHz to 300 GHz, are a segment of the electromagnetic spectrum [105]. Microwave heating (MH) is a rapid and effective heat treatment method. MH can preserve more nutrients in the product compared to infrared heating and moist heat technologies and is commonly utilized in the food industry [106]. In order to speed up the hydrogen-bonding interactions between proteins, lower the free thiol content, and quickly produce protein hydrolysis products, microwave heating typically uses electromagnetic energy to create intermolecular collisions through the migration of ionic particles or the rotation of dipole particles. Proper MH treatment promotes the formation of β-sheets while disrupting α-helices, β-helices, and random coiling. The use of microwave treatment significantly increased laccase’s energy storage modulus and water-holding capacity and generated soy protein isolate gels and soy protein isolate/wheat gluten gels caused by microbial transglutaminase [107].

## 4. Bioactive Substance Delivery

Microscopic molecules known as bioactive compounds (bioactives) are present in varying amounts in a variety of meals. Many dietary sources, such as oils, vegetables, fruits, nuts, legumes, and seafood, provide bioactive and useful substances such as vitamins, minerals, taste and fragrance compounds, peptides, flavonoids, carotenoids, probiotics, and polyphenols [68,108,109]. In addition to improving human health, they also carry out a variety of biological tasks in the body, including decreasing cholesterol, regulating the immune system, performing anticancer, anti-inflammatory, antioxidant, antibacterial, and antiallergic effects [110,111,112]. Although scientists have attempted to use these bioactives in the manufacturing and processing of different products, their instability prevents further use. They have a low capacity for sustainability and bioavailability, are very susceptible to oxidation, and are poorly soluble in water. They list the main drawbacks that prevent their application in the food, cosmetic, and pharmaceutical industries [113]. Numerous ways have been implemented to surmount these challenges. Encapsulation technology is extensively employed due to its ability to optimize the preservation of functionality [114]. Because of their high encapsulation efficiency, biocompatibility, cost-effectiveness, and ecologically beneficial properties, hydrogels are being used more and more as delivery and encapsulating agents (as shown in Table 2). Because of their porous character, which comes from a three-dimensional structure where crosslinked polymers offer large interstitial gaps that are heavily occupied by water, these features are possible. Numerous minerals and bioactive compounds can also be sequestered by these interstitial gaps. Because of this, these areas can be leveraged to get around some of the challenges associated with adding healthy nutrients to food, such as poor solubility, low thermal and chemical stability, and unappealing flavor and sensory profiles. Bioactive substances are protected from outside environmental impacts during production, storage, and after ingestion by being encapsulated in hydrogels.

### 4.1. Polyphenols

Many natural plants include polyphenols, which are organic compounds with phenolic hydroxyl groups that are utilized to treat cancer, inflammation, and bacteria [123,124]. Epicatechin gallate (ECG), gallic acid (GA), dopamine (DA), and tannic acid (TA) are examples of common natural polyphenols. Polyphenols are now the most significant antioxidant in the human diet because of their reducibility and extensive use in natural foods and beverages [125]. In actuality, free polyphenols’ physicochemical characteristics are unstable, their dispersion in water is inadequate, and their function is impacted by changes in the gastrointestinal tract as well as environmental factors like light, air, and temperature. As a transport system for polyphenols, the hydrogel made from protein and polysaccharide as raw materials shields the compounds from air, heat, light, and pH levels, preventing structural damage during processing and storage and enhancing their bioavailability.

Quercetin is a flavonoid that has therapeutic qualities such as antioxidant, anti-inflammatory, anticancer, and cardioprotective capabilities, but low solubility and physicochemical instability make it difficult to be absorbed and utilized by the human body. In order to stop quercetin from degrading in the environment, Cao et al. created zeinolysin/soy polysaccharide SSPS composite colloidal nanoparticles at pH 4.0 using a simple antisolvent precipitation technique, making sure that no aggregation or precipitation took place at pH values between 2.0 and 8.0. Additionally, they demonstrated relative stability at higher temperatures and ionic strengths. Because of the SSPS coating, the zeinolysin nanoparticles’ surface hydrophobicity decreased over a wide pH range. The hydrophobic quercetin was successfully confined by the production of the zeinolysin/SSPS nanoparticles. After SSPS coating, the encapsulation effectiveness increased dramatically to 82.5%, compared to 59.2% for untreated zeinolysin. Furthermore, quercetin’s ABTS-scavenging efficiency and photochemical stability in maize-alcohol-solubilized protein/SSPS composite nanoparticles were significantly enhanced. In food and medication formulations, zeinolysin/SSPS composite nanoparticles can be used as an all-natural way to distribute bioactive substances [115]. Using an ionic gelation technique, Marudova et al. successfully loaded quercetin into casein/chitosan gel polyelectrolyte complexes. The properties of the complexes are strongly influenced by the mass ratio of the polymers. The complexes had nanoscale dimensions with an average diameter of around 400 nm at casein/chitosan charge ratios of 1:1, 2:1, and 4:1; however, when more casein was added, their diameters dramatically increased to several micrometers. The casein/chitosan charge ratio affected the complexes’ yield; with more casein, it increased from 37.5% to 72.5%. With loading efficiencies above 95%, quercetin is expected to be trapped in the hydrophobic area of casein micelles due to its hydrophobic characteristics [116].

**Figure 3:**
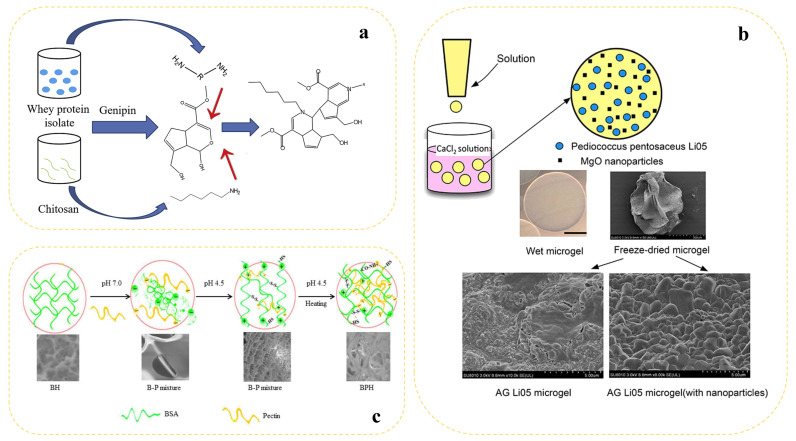
(**a**) Schematic preparation of whey protein–chitosan composite hydrogels loaded with curcumin [117]. (**b**) Schematic diagram of microgel preparation loaded with *Pediococcus pentosaceus* Li05 (the blue dots represent probiotics) [121]. (**c**) Schematic preparation of bovine serum albumin–citrus pectin hydrogels providing vitamin C and SEM characterization of their microstructures [119].

Curcumin is a phenolic molecule generated from turmeric with good functional activities such as anti-inflammatory and anti-cancer effects. Nonetheless, it has inadequate water solubility and bioavailability. Condensed whey protein isolate (WPI)–chitosan (CS) composite hydrogels were created by Xu et al. using the natural agent genipin (GP) for cross-linking in order to address this problem (Figure 3a). The composite gel with 1% CS demonstrated the highest storage modulus, significantly outperforming the other gels, according to the frequency scans. The gel outperformed the other gels in terms of hardness (4000 N), water-holding capacity (100%), and denaturation temperature (93.7 °C). Furthermore, the resulting gel had a 70.4% cross-linking degree, making it difficult for trypsin and pepsin to dissolve. Curcumin release studies, on the other hand, demonstrated that the composite hydrogel had a significant advantage in terms of continuous curcumin release, which eventually reached 7% at 4 h and persisted. Consequently, WPI–CS hydrogel could be a helpful delivery method for controlled bioactive release in functional foods and pharmaceutical applications [117].

Epigallocatechin gallate (EGCG) is a phenolic catechin with numerous advantageous health qualities, including antioxidant and anticancer effects. The polyhydroxy configuration of catechins renders them unstable at neutral and alkaline pH levels. Wang et al. reported a composite hydrogel made from many biocompatible carboxymethyl konjac glucomannan (CKGM)/gelatin (G)/tannic acid (TA)-functionalized nano-hydroxyapatite (TA@n-HA) to boost the bioavailability of EGCG. This hydrogel has shown good pH sensitivity and biodegradability. Various pH values resulted in various EGCG release patterns, which enhanced EGCG’s encapsulation and bioavailability [118].

### 4.2. Vitamin Class

Vitamins are defined as a group of essential micronutrients that the body cannot synthesize. They are divided into fat-soluble vitamins (A, D, E, and K) and water-soluble vitamins (such as folic acid) [126]. Serious illnesses like scurvy and night blindness can result from vitamin deficiencies. Some active ingredients, on the other hand, are either produced in the body in extremely minute quantities or are only available through external intake. As a result, vitamins must be taken as dietary supplements. Because vitamins are chemically reactive and sensitive to external conditions including light, pH, temperature, and oxygen, they break down readily during food processing and storage. Because of their special three-dimensional network, hydrogels can offer an environment that is appropriate for their load, shield embedded substances like vitamins from outside environmental stimuli, and achieve controlled release at particular gastrointestinal tract locations, all of which increase the stability of the gel. Thus, to preserve and administer vitamins, hydrogels made from food-grade biopolymers (proteins and polysaccharides) have been employed. Xiong et al. used the natural polymers bovine serum albumin (BSA) and pectin from citrus fruit peels to create a novel hydrogel (BSA–pectin hydrogel, BPH) via a self-assembly approach, as shown in Figure 3c. The electrostatic and covalent interactions between BSA and pectin were shown to be the primary pathways for the synthesis of BPH by the rheological characterization of BPH and the conformational structure of the gel. The hydrogel has a narrow size distribution (polydispersity index < 0.06) and a strong, stable three-dimensional network structure. With Vc as the active agent, BPH had a noticeably higher EE (65.31%) and better Vc retention (73.95%) after 10 weeks of storage. Consequently, in both food and non-food applications, BPH might be a viable delivery method to improve the stability and bioavailability of functional chemicals [77]. In order to improve the controlled release of vitamin E (VE) and the bioavailability, Li et al. synthesized three-dimensional, nanocomposite, highly absorbent, ductile, and bioadhesive hydrogel HGCs using the chemical cross-linking agent poly (ethylene glycol) diglycidyl ether (PEGDGE). In a soybean soluble-polysaccharide (SSPS) polymer matrix, *β*-cyclodextrin (*β*-CD) was assembled into polynuclear units. Through host–guest interactions, the *β*-CD cavities served as carriers for vitamin E (VE). They had a loading capacity of 16.04% and an encapsulation efficiency of 79.10%. Furthermore, HGNCs demonstrated a sustained release (more than 230 h) of VE in vitro along with excellent solubilization adsorption. In comparison to free VE solutions, the relative pharmacological bioavailability of VE from VE-loaded HGNCs was enhanced (up to 7.5-fold) [119]. Co-assemblies of the isoproteins lactoferrin (LF) and *β*-lactoglobulin (BLG) were investigated for their potential as vitamin B9 (B9) biocarriers. Different B9/protein mixing ratios were used to create B9–LF–BLG co-assemblies, which were then evaluated using phase-contrast imaging and turbidimetric analysis. With an ideal capture of about 10 mg B9/g protein, the B9–LF–BLG coalescents demonstrated enhanced performance as B9 biocarriers. As a result, this co-assembly shows great promise as a vitamin biocarrier [120].

### 4.3. Probiotic Class

Probiotics are live microbial dietary supplements that are believed to play a vital role in sustaining health due to their many positive effects on the host system when taken in suitable numbers [127,128]. Clinical investigations have demonstrated the potential of oral probiotics to cure a variety of clinical conditions, including lactose intolerance, diarrhea, and some allergies. Additionally, probiotics are effective in immunomodulation and in increasing the bioavailability of zinc, calcium, copper, iron, manganese, and phosphorus [129]. However, for consumed probiotics to positively impact host health and physiology, they must make it through the stomach and into the intestine in sufficient quantities. The severe acidity of the human gastrointestinal tract is the main barrier to ingested bacteria’s survival. Furthermore, during drying and storage, environmental factors such oxygen, heat, and humidity may cause lipid oxidation, cell wall disintegration, or changes to the cell membrane [130,131]. As a result, polysaccharide/protein hydrogels are efficient probiotic carriers that improve cellular defense against harmful gastrointestinal disorders, encourage gradual intestinal cell release, and increase the stability and viability of probiotic cells under various processing and storage temperatures and humidity levels [132]. Using Lactobacillus casei, Yang et al. successfully created a bilayer filipin/sodium alginate hydrogel wound scaffold (L-SF/SA) with a porous structure and advantageous swelling properties. This created the ideal environment for Lactobacillus casei adherence and viability. Concurrently, adding Lactobacillus casei to the scaffold gave it exceptional antibacterial activity. Through the IRE1/XBP1 pathway, we demonstrated that L-SF/SA gel can reduce TGF-*β*1-induced fibrosis, accelerating wound healing and reducing the formation of scars. The L-SF/SA scaffold reduced the amount of germs in infected wounds, promoted the polarization of macrophages, reduced inflammation, increased angiogenesis, and accelerated the regeneration of hair follicles. Probiotic-functionalized SF/SA scaffolds are therefore perfect dressings for the future treatment of chronic wounds and skin regeneration since they have a great deal of potential to reduce wound infections and enhance the quality of healing [105]. Figure 3b illustrates that Li et al. used magnesium oxide (MgO) nanoparticles (NPs) in an alginate–gelatin microgel to enclose a model probiotic (Pediococcus pentosaceus Li05). The morphology and surface characteristics of the encapsulated system were investigated using transmission electron microscopy (TEM) and atomic force microscopy (AFM), which showed that probiotics and nanoparticles were effectively incorporated into the spherical microgels. To evaluate the probiotics’ ability to survive, the microgels were subjected to a variety of conditions, including heat treatment, gastrointestinal transit, and prolonged storage in an aerobic environment. The encapsulation of probiotics greatly increased their viability under various circumstances. MgO-loaded microgels may be a useful encapsulation and delivery method to increase the effectiveness of oral probiotics by protecting them from unfavorable storage and gastrointestinal environment conditions [121]. Liu et al. used ultrasonication and enzyme induction to create a composite hydrogel comprising soy protein SPI and pectin CP coupler. After 20 min of ultrasonication, the SPI/HMP coupling’s particle size decreased and its hydrophobicity rose. Following several ultrasonication treatments, changes in the secondary structure and hydrophobic amino acid residues of SPI and SPI/CP coupling were shown via FT-IR and fluorescence spectroscopy. The structural properties were significantly altered by sonication and the addition of pectin, which produced a porous structure. The findings on rheological parameters showed that a continuous network structure was formed by the SPI/CP hydrogels. The connections’ surface hydrophobicity was enhanced by sonication, which raised the gel’s strength and water-holding capacity. The hydrogel samples were sonicated for 20 min, which improved Lactobacillus plantarum’s ability to survive simulated gastrointestinal digestion and encouraged intestine-targeted release. Hydrogels may also improve probiotic viability when exposed to UV light. These discoveries could lay the theoretical groundwork for the development of novel natural delivery strategies for proteins and polysaccharides [122].

## 5. Conclusions and Future Research Perspectives

Food gels are widely employed in the encapsulation, distribution, and administration of bioactive compounds because of their distinctive three-dimensional network structure, mechanical properties that can be adjusted, and the biocompatibility of polysaccharide and protein, which are the primary hydrogel raw materials. As a result, this work described the new preparation techniques (high pressure, ultrasonic, microwave, etc.) as well as the conventional techniques of polysaccharide and protein gelation (temperature, pH, ionic strength, etc.). The most recent developments in food gels’ ability to transport bioactive compounds are also covered, offering a fresh concept for the future development and use of polysaccharide–protein complex hydrogels in food formulation and bioactive delivery. Nevertheless, there are still certain issues and difficulties with real-world implementation. For instance, (1) gel structure design may continue to play a significant role in the creation of gel-related foods in future food research. A layered gel structure with lipophilic bioactive components is anticipated to be produced by combining the rheological and interface properties of polysaccharides. This opens the door for additional application design of the polysaccharide–protein hybrid system in gel-related foods. (2) The need to create personalized functional foods, such as particular foods for people with diabetes, obesity, and cardiovascular disease, stems from the growing demand for healthy foods. (3) It is necessary to investigate the metabolism of food gels in the body because some of them can be immediately absorbed by the body but cannot be broken down or digested. Furthermore, food gel safety is a matter that requires investigation in both design and study.

## Figures and Tables

**Table 1:** Preparation of various protein hydrogels for food and biomedical applications.

	Other Compound Ingredients	Crosslinking/Preparation Strategies	Characteristic	Reference
Soy protein		heating	Enhanced gel properties	[39]
	reducing sugar	heating	higher WHC and lower gel hardness	[40]
		phosphorylation modification	G′ and G″ increase and Show significant elastic behavior	[41]
Pea protein		pH-induced	Thermally reversible	[42]
		pH-induced	Good elasticity and high water holding capacity	[43]
		freezing and thawing method	Minimum dehydration contraction level of 15%	[43]
		pH-induced	Excellent gel water retention and stability	[44]
		SDS and ionization	Dense and stable adhesive	[45]
Whey protein		heating and pH-induced	Higher iron release	[46]
Whey protein	gelatin	enzyme-induced	Greater hardness and solubility	[47]
Pea protein	corn alcohol protein	enzyme-induced	High gel strength and water holding capacity	[48]
Soy protein	cellulose		Higher viscosity and modulus	[49]
Soy protein	glucose	ultrasound induced	Encapsulation efficiency of up to 65.9%	[50]
Corn Alcohol Protein	alginate	ionization	Improved light and heat stability of curcumin	[51]

**Table 2:** Application of various polysaccharide/protein hydrogels in the delivery of bioactives.

Classification	Bioactive Substance	Gel Composition	Characteristic	Reference
Polyphenol	Quercetin	Corn alcohol soluble protein/soybean polysaccharide	Quercetin encapsulation efficiency increased to 82.5%, with significant enhancement of photochemical stability and ABTS-scavenging ability.	[115]
		Casein/chitosan	Load efficiency of 95% or more	[116]
	Curcumin	Whey protein isolate (WPI)—Chitosan (CS)	The resulting gel had a cross-linking degree of 70.4%, was not easily hydrolyzed by pepsin and trypsin, had a curcumin release rate of 7% at 4 h, and continued to be released.	[117]
	Gallocatechin Gallate	Carboxymethyl konjac glucomannan (CKGM)/gelatin (G)/tannic acid (TA) functionalized nano-hydroxyapatite	Hydrogels have good biodegradability and pH sensitivity.	[118]
Vitamins	Vitamin C	Bovine serum albumin (BSA) and citrus peel pectin	Has a high higher EE (65.31%) and enhanced Vc retention after 10 weeks of storage (73.95%).	[77]
	Vitamin E	*β*-cyclodextrin, soybean soluble polysaccharide	Ve package efficiency and load capacity are 79.10% and 16.04%, respectively.	[119]
	Vitamin B9	The isoprotein beta-lactoglobulin (BLG) and lactoferrin (LF)	The B9–LF–BLG condensate showed higher performance as a B9 biocarrier with an optimal capture of ≈10 mg B9/g.	[120]
Probiotics	Lactobacillus Casei	Silk proteins/sodium alginate	Probiotic-functionalized SF/SA scaffolds have great potential for controlling wound infection and improving the quality of healing and are ideal dressings for the future treatment of chronic wounds and skin regeneration.	[105]
	Pediococcus Pentosaceus Li05	Alginate–gelatin	Encapsulation of probiotics significantly enhanced their viability under these different conditions.	[121]
	Lactobacillus Plantarum	Soy protein and pectin	Effectively improves the survival of Lactobacillus plantarum during simulated gastrointestinal digestion and promotes intestinal targeted release.	[122]
